# Chest CT Computerized Aided Quantification of PNEUMONIA Lesions in COVID-19 Infection: A Comparison among Three Commercial Software

**DOI:** 10.3390/ijerph17186914

**Published:** 2020-09-22

**Authors:** Roberto Grassi, Salvatore Cappabianca, Fabrizio Urraro, Beatrice Feragalli, Alessandro Montanelli, Gianluigi Patelli, Vincenza Granata, Giuliana Giacobbe, Gaetano Maria Russo, Assunta Grillo, Angela De Lisio, Cesare Paura, Alfredo Clemente, Giuliano Gagliardi, Simona Magliocchetti, Diletta Cozzi, Roberta Fusco, Maria Paola Belfiore, Roberta Grassi, Vittorio Miele

**Affiliations:** 1 Division of Radiodiagnostic, “Università degli Studi della Campania Luigi Vanvitelli”, 80138 Naples, Italy; roberto.grassi@unicampania.it (R.G.); salvatore.cappabianca@unicampania.it (S.C.); fabrizio.urraro@unicampania.it (F.U.); giuliana.giacobbe@unicampania.it (G.G.); gaetanomaria.russo@unicampania.it (G.M.R.); assunta.grillo@unicampania.it (A.G.); alfredo.clemente@unicampania.it (A.C.); simona.magliocchetti@unicampania.it (S.M.); mariapaola.belfiore@unicampania.it (M.P.B.); robertagrassi89@gmail.com (R.G.); 2 Department of Medical, Oral and Biotechnological Sciences—Radiology Unit “G. D’Annunzio” University of Chieti-Pescara, 66100 Chieti, Italy; beatriceferagalli@hotmail.com; 3 Laboratory Medicine Unit, ASST Bergamo Est, 24068 Seriate, Italy; al.montanelli@asst-bergamoest.it; 4 Department of Radiology, ASST Bergamo Est, 24068 Seriate, Italy; patellig@yahoo.it; 5 Radiology Division, “Istituto Nazionale Tumori IRCCS Fondazione Pascale—IRCCS di Napoli”, 80131 Naples, Italy; v.granata@istitutotumori.na.it; 6 Diagnostic Imaging Unit, “Azienda Ospedaliera di Rilievo Nazionale Giuseppe Moscati”, 83100 Avellino, Italy; angela.delisio@libero.it (A.D.L.); cesare.paura@hotmail.it (C.P.); giuliano.gagliardi@hotmail.it (G.G.); 7 Division of Radiodiagnostic, Azienda Ospedaliero-Universitaria Careggi, 50139 Firenze, Italy; dilettacozzi@gmail.com (D.C.); vmiele@sirm.org (V.M.)

**Keywords:** COVID-19, computed tomography, computer-aided quantification

## Abstract

Purpose: To compare different commercial software in the quantification of Pneumonia Lesions in COVID-19 infection and to stratify the patients based on the disease severity using on chest computed tomography (CT) images. Materials and methods: We retrospectively examined 162 patients with confirmed COVID-19 infection by reverse transcriptase-polymerase chain reaction (RT-PCR) test. All cases were evaluated separately by radiologists (visually) and by using three computer software programs: (1) Thoracic VCAR software, GE Healthcare, United States; (2) Myrian, Intrasense, France; (3) InferRead, InferVision Europe, Wiesbaden, Germany. The degree of lesions was visually scored by the radiologist using a score on 5 levels (none, mild, moderate, severe, and critic). The parameters obtained using the computer tools included healthy residual lung parenchyma, ground-glass opacity area, and consolidation volume. Intraclass coefficient (ICC), Spearman correlation analysis, and non-parametric tests were performed. Results: Thoracic VCAR software was not able to perform volumes segmentation in 26/162 (16.0%) cases, Myrian software in 12/162 (7.4%) patients while InferRead software in 61/162 (37.7%) patients. A great variability (ICC ranged for 0.17 to 0.51) was detected among the quantitative measurements of the residual healthy lung parenchyma volume, GGO, and consolidations volumes calculated by different computer tools. The overall radiological severity score was moderately correlated with the residual healthy lung parenchyma volume obtained by ThoracicVCAR or Myrian software, with the GGO area obtained by the ThoracicVCAR tool and with consolidation volume obtained by Myrian software. Quantified volumes by InferRead software had a low correlation with the overall radiological severity score. Conclusions: Computer-aided pneumonia quantification could be an easy and feasible way to stratify COVID-19 cases according to severity; however, a great variability among quantitative measurements provided by computer tools should be considered.

## 1. Introduction

The spread of severe acute respiratory syndrome coronavirus 2 (SARS-CoV-2) has already assumed pandemic proportions, affecting over 100 countries in a few weeks. A global response is needed to prepare health systems worldwide [1,2]. Covid-19 can be diagnosed both on chest X-ray and on computed tomography (CT). Asymptomatic patients may also have lung lesions on imaging. CT investigation in patients with suspicion Covid-19 pneumonia involves the use of the high-resolution technique. Artificial intelligence (AI) software for quantification of Pneumonia Lesions has been employed to facilitate CT diagnosis [3,4,5].

Several radiological organizations do not recommend CT as a primary diagnostic/screening tool for COVID-19 [6,7,8,9] or have excluded CT findings from its diagnostic criteria [10]. However, the viral pneumonia diagnosis on chest CT plays an important role in the management of patients with suspected SARS-CoV-2 infection, especially as anticipation of mild invasive ventilation has been proven effective in reducing the severity of pneumonia [11,12], in the absence of proven therapies for the treatment of COVID-19. Radiologists focus on main CT findings (GGO: ground-glass opacity and consolidation), and lesion distribution (left, right, or bilateral lungs) [10]. Bilateral distribution of GGOs, with or without consolidation, in posterior and peripheral lungs, was initially described as a characteristic feature of COVID-19 [11,12].

Machine learning-based technologies and computer tools are playing a substantial role in the COVID-19 pandemic. Experts are using machine learning to study the virus, test potential treatments, diagnose individuals, analyze the public health impacts, and more. Computer software could be useful categorizing the disease into different severities with quantitative, objective assessments of the extent of the lesions [13,14,15,16].

Computer tools have recently been proposed for the recognition of lung lesions from Covid-19 on CT examination, many of which are Chinese [17,18,19]. However, many of them are not recognized as medical devices nor do they have the CE marking. Furthermore, they have been tested on thousands of cases of COVID-19 but not equally on as many cases of non-COVID-19 coronavirus, affecting their ability to make a differential diagnosis. The recognition of interstitial pneumonia lesions on a chest CT scan does not pose any difficulties and therefore the role of computer tools remains limited to the numerical quantization of the lesions and their distribution.

Proof of principle of the diagnostic capability of deep learning methods from CT images to screen for COVID-19 has been recently demonstrated by Wang et al. [20] on 1119 CT images of pathogen-confirmed COVID-19 cases versus typical viral pneumonia. The accuracy and applicability of deep learning for screening COVID-19 from CTs have however been questioned, based on concerns of the radiologists’ association and given the impact of selection bias reported in first published results.

In this manuscript, we presented three commercial tools used to codify lung volumes on CT in Covid-19 patients (Thoracic VCAR software, GE Healthcare, Chicago, IL, USA; Myrian, Intrasense, France; InferRead, InferVision Europe, Wiesbaden, Germany).

## 2. Materials and Methods

### 2.1. Patient Characteristics

This retrospective study included patients enrolled by “Bergamo Est” hospital, Bergamo, by “AORN Giuseppe Moscati”, Avellino and “University Vanvitelli”, Napoli. In relation to the ongoing epidemic emergency, the institutional local review boards waived written informed consent for this retrospective study that evaluated de-identified data and involved no potential risk to patients. The population included 162 patients (57 women and 105 men; 67 years of median age-range, 26–93 years) subjected to the nucleic acid amplification test of the respiratory tract or blood specimens using reverse transcription real-time fluorescence polymerase chain reaction test with confirmation of COVID-19 infection, between 23 February 2020, and 31 March 2020. The virus investigation for etiological diagnosis was executed by the current gold standard test (RT-PCR).

### 2.2. CT Technique

Chest CT scan was performed at the time of patient admission in a hospital with three CT scanners: two scanners with 128 slices (Ingenuity of Philips, Amsterdam, Netherlands and Revolution of GE Healthcare, Chicago, IL, USA), one CT scanner with 64 slices (Toshiba Aquilion 64 Slices, Tokyo, Japan). CT examinations were performed with the patient in the supine position in breath hold during and inspiration using a standard dose protocol, without contrast intravenous injection. The scanning range was from the apex to the base of the lungs. The tube voltage and the current tube were 120 kV and 100–200 mA, respectively. All data were reconstructed with a 0.6–1.0 mm increment. The matrix was 512 mm × 512 mm. Images were reconstructed using a sharp reconstruction kernel for parenchyma and viewed at window settings optimized for the assessment of the lung parenchyma (window width: 1600 HU; window level: 600 HU).

### 2.3. CT Post-Processing

DICOM data were transferred into a PACS workstation and CT images were evaluated using three clinically available computer tools: Thoracic VCAR software (GE Healthcare, Chicago, Illinois, United States); Myrian software (Intrasense, France); InferRead tool (InferVision Europe, Wiesbaden, Germany). Table 1 reports a comparison among evaluated commercial software based on the provided functionalities.

#### 2.3.1. Post-Processing with Thoracic VCAR Software

**Figure 1:**
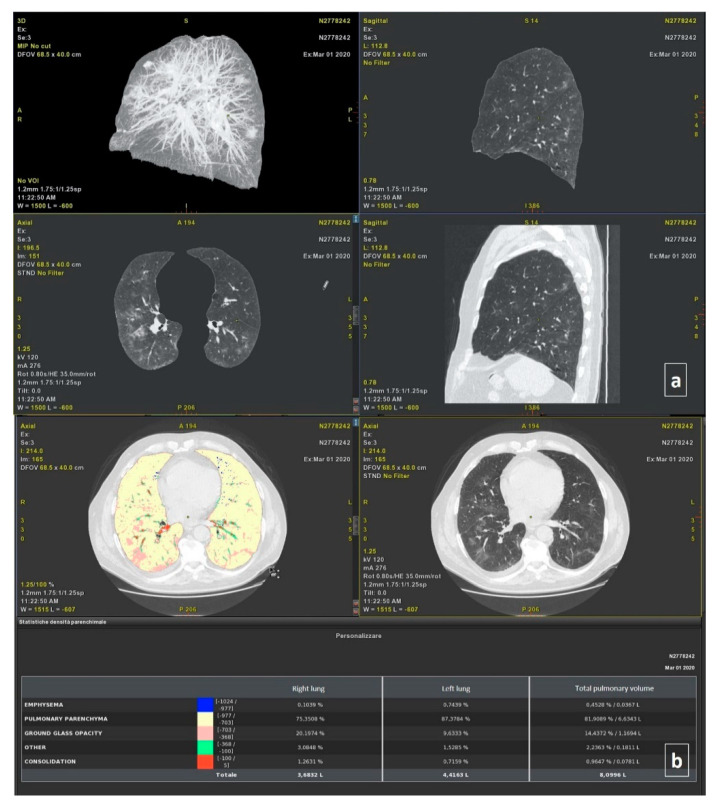
Automatic Segmentation of Thoracic Disease by COVID-19 using the Thoracic VCAR Tool of General Electric Healthcare: (**a**) 3D axial and sagittal plane reconstruction; (**b**) Density analysis of parenchyma. This case had bilateral and diffuse ground-glass opacity (GGO) and consolidations in multiple lobes.

Thoracic VCAR software is a CE marked medical device designed to quantify pulmonary emphysema in patients with Chronic Obstructive Pulmonary Disease. The software provides automatic segmentation of the lungs and automatic segmentation and tracking of the airway tree. It provides the classification of voxels based on Hounsfield Units and a color-coded display of the thresholds within a segmented region. We analyzed the CT scans of patients with confirmed COVID-19 pneumonia by pre-setting a threshold value of Hounsfield Units to obtain a segmentation of both lungs and a quantitative evaluation of Emphysema (−1024/−977; blue), Healthy residual lung parenchyma (−976/−703; yellow), GGO (−702/−368; pink) and consolidation (−100/5; red). Finally, volumes for both the right and left lung were calculated (Figure 1).

#### 2.3.2. Post-Processing with Myrian Software

The Myrian solution developed by Intrasense teams automatically provides an objective measurement of the impairment and the available pulmonary reserve of patients, allowing the identification of healthy and pathological areas (ground glass and consolidations areas). These elements provide the pulmonary reserve as well as a density histogram over a complete pulmonary volume. Moreover, the system automatically generates structured diagnosis reports and follow-up reports for pneumonia cases.

**Figure 2:**
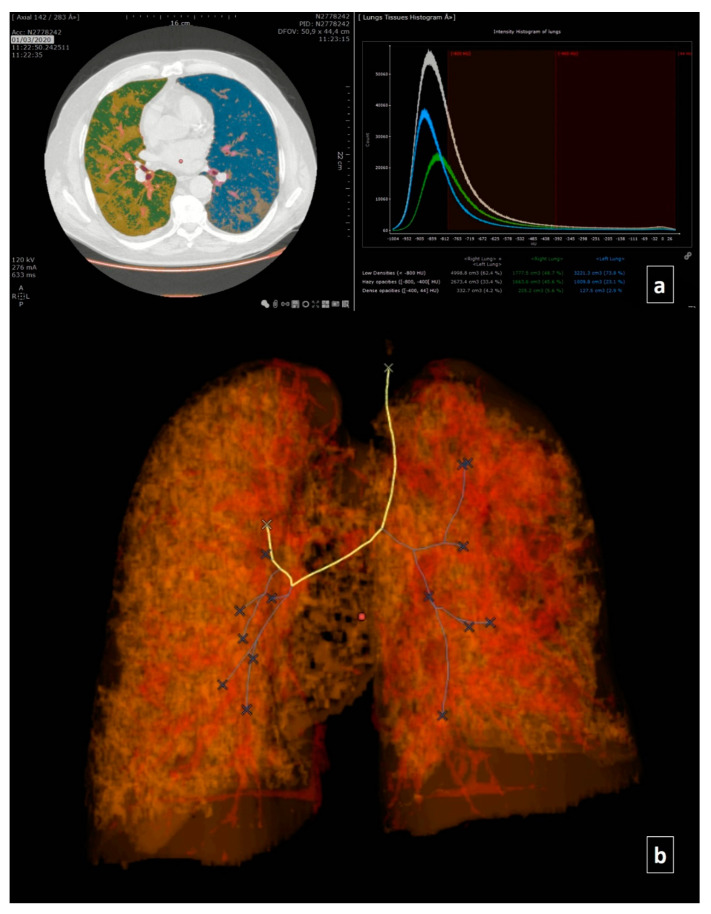
The same case of Figure 1. Automatic Segmentation of Thoracic Disease by COVID-19 using the Myriam Tool of Intrasense: (**a**) Intensity histogram of lungs; (**b**) 3D reconstruction.

We analyzed using Myrian software the CT scans and registered the healthy parenchyma (−1000/−801), GGO (−800/−400), and consolidation volumes (−399/69) (Figure 2).

#### 2.3.3. Post Processing with InferRead Software

**Figure 3:**
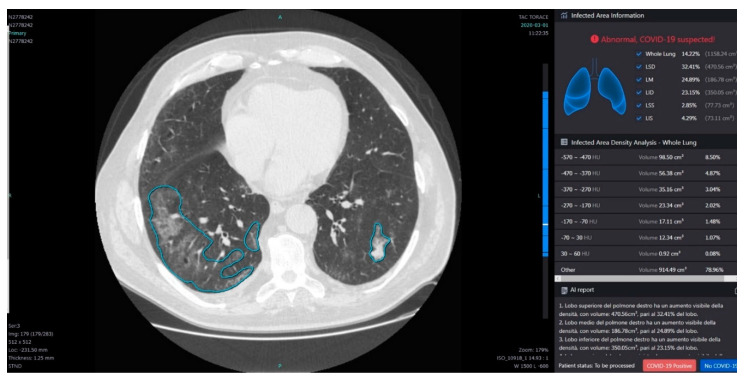
The same case of Figure 1. Automatic Segmentation (Blue shaped infected area density analysis) of Thoracic Disease by COVID-19 using the InferRead Tool of InferVision.

The InferRead system shows density distribution via histograms and calculates the percentage of the lung volume occupied by each lobe and the percentage of the volume with different Hounsfield unit density considering the following ranges: −570/−470; −470/−370; −370/−270; −270/−170 (%); −170/−70; −70/30; 30/60 and other. Moreover, the tool provides a follow-up management tool to enable online follow-up for high-risk patients during quarantine, which allows remote tracking of patient status and help clinicians arrange further exams. Moreover, the system automatically generates structured diagnosis reports and follow-up reports for pneumonia cases. We analyzed using InferRead system software the CT scans and registered the volumes with different density (Figure 3) and then we calculated the GGO volume (summing the volumes in these ranges −570/−470; −470/−370; −370/−270), consolidation area (summing the volumes in these ranges −170/−70; −70/30; 30/60) and healthy parenchyma volume (lung volume remaining).

### 2.4. Radiologists Analysis

Radiologists attributed for each lung looking the CT images at the pulmonary involvement by disease a severity score using a scale of 5 levels (none, mild: ≤25% of involvement, moderate: 26–50% of involvement, severe: 51–75% of involvement and critic involvement: 76–100%). Moreover, an overall radiological severity score was obtained summing the scores for each lung and then considering a mild severity a score ≤2, moderate 3–4, severe 5–6, and critic 7–8. Two radiologists with more than 10 years of thoracic-imaging analysis experience evaluated the severity of images in a double-blind manner. Another, more experienced, radiologist resolved any disagreement between the two radiologists. 

### 2.5. Statistical Analysis

Continuous data were expressed in terms of median value and range. Mann–Whitney test and Kruskal–Wallis test were used to verify differences between groups. Spearman correlation coefficient and intraclass correlation coefficient were used to analyze the correlation and variability among quantitative measurements generated by different computer tools and between radiological severity score obtained by the radiologists and quantitative results generated by the computer software.

*p*-value < 0.05 was considered significant for all tests.

All analyses were performed using IBM SPSS Statistics 24 (IBM, Armonk, NY, USA).

## 3. Results

Thoracic VCAR software was not able to perform volumes segmentation in 26/162(16.0%) cases, Myrian software in 12/162 (7.4%) patients while InferRead software in 61/162(37.7%) patients.

The ICC showed great variability among the quantitative measurements of the residual healthy lung parenchyma volume, GGO, and consolidations volumes obtained by different computer tools (Table 2). The lowest variability was reported for GGO volume.

The Spearman correlation analyses (Table 3) showed a moderate correlation between lesion percentage determined by Thoracic VCAR and Myrian software (ranged from 0.54 to 0.78, all *p* < 0.05) while a low or mild correlation between lesion percentage determined by Thoracic VCAR and InferRead software was obtained (ranged from 0.34 to 0.50, all *p* < 0.05) and a low or mild correlation between lesion percentage determined by Myrian and InferRead software (ranged from 0.31 to 0.61, all *p* < 0.05).

The lung volumes quantified using the ThoracicVCAR tool were significantly lower in those with severe disease than in those without severe disease (*p* < 0.05) for the residual healthy lung parenchyma and GGO volumes (Table 4). Instead using Myrian software only residual healthy lung parenchyma and consolidation volumes showed differences statistically significant among patients with different severity scores (Table 5) while using the InferRead tool only residual healthy lung parenchyma and GGO volumes showed differences statistically significant (Table 6).

Overall radiological severity score was moderately correlated with the residual healthy lung parenchyma volume obtained by ThoracicVCAR or Myrian software (Spearman coefficient = 0.70–0.74), with GGO area obtained by the ThoracicVCAR tool (Spearman coefficient = 0.65) and with consolidation volume obtained by Myrian software (Spearman coefficient = 0.65) (Table 4 and Table 5). Instead, low correlations were reported among the overall radiological severity score and each quantitative measurement obtained by InferRead software (Table 6).

## 4. Discussions

Several publications have described X-rays role and CT imaging features in patients affected by COVID-19, the evolution of these features over time, and the radiologist’s performance to differentiate COVID-19 from other viral infections [10,11,12,13]. These studies have shown that typical CT findings of COVID-19 infection occur with two different patterns: peripheral, bilateral GGO with or without consolidation or intralobular lines (“crazy paving”); multifocal GGO of rounded morphology with or without consolidation or “crazy paving” [20]. The less typical pattern is characterized by non-peripheral non-rounded GGO with multifocal, diffuse, perihilar, or unilateral distribution, with or without consolidations [21,22].

Several methods of disease extent quantification at chest CT using machine learning and AI tools have been proposed, including the extent of emphysema, GGO, and consolidation [23,24,25,26,27,28,29,30]. A visual semi-quantitative quantification of the disease extent at CT correlated with clinical severity [31].

Colombi et al. [32] reported that in patients with confirmed COVID-19 pneumonia, visual or software quantification the extent of CT lung abnormality were predictors of Intensive Care Unit (ICU) admission or death. They reported that the proportion of well-aerated lung assessed by chest CT was associated with better prognosis independent of other clinical parameters. Gozes et al. [19] used 2D and 3D deep learning models to explore AI-based automated CT image analysis tools for detection, quantification, and tracking of Coronavirus. One hundred and six COVID-19 chest CT scans and 99 normal ones were used to find potential COVID-19 thoracic CT features and to evaluate the progression of the disease in patients over time, generating a quantitative score.

At the best of our knowledge, this manuscript is the first with the aim to compare different computer tools for quantification in COVID-19 patients of pneumonia lesions on chest CT.

We demonstrated that there was great variability among the quantitative measurements obtained by different commercial computerized tools. Moreover, we reported differences statistically significant among volumes of residual healthy lung parenchyma, GGO, and consolidation considering the overall radiological score of patients with severe disease respect to those without severe. In addition, we reported that the overall radiological severity score was moderately correlated with the residual healthy lung parenchyma volume obtained by ThoracicVCAR or Myrian software, with the GGO area obtained by the ThoracicVCAR tool and with consolidation volume obtained by Myrian software. Instead, InferRead software had a low correlation with the overall radiological severity score.

Therefore, considering our results, the ThoracicVCAR and Myrian tools seem to be the most effective and easiest software programs to provide automatic quantitative measurement in COVID-19 patients because it provides a semi-automatic and fast segmentation of lesions; a visualization of pathological lung areas (ground-glass opacities, crazy paving, consolidations, emphysematous areas). Therefore, these tools can be used in clinical practice to assist radiologists diagnoses.

An ideal software for COVID-19 should have automatic recognition of internal lung fields; the possibility to exclude airways and pulmonary vessels; automatic recognition of increased caliber peripheral pulmonary vessels; automatic recognition of increased caliber (over 2.9 cm) pulmonary artery; the possibility of calculating the percentage of emphysematous parenchyma, GGO, consolidation, and well ventilated residual lung parenchyma; the distinct percentage for lobes, lungs and total; the possibility of reporting these percentages values in the reference without copying them; the possibility to memorize lesions volume automatic quantification for possible comparison with a subsequent examination of the same patient.

The limitations of this study included the retrospective nature of the study and the sample size having determined the great variability of three computer tools in lung volumes quantification.

In the future, examining the correlation between quantitative CT parameters and clinical symptoms and laboratory indices would be useful for guiding clinical decision-making.

In summary, computer-aided quantification could be an easy and feasible way to stratify patients according to disease severity by COVID-19; however, a great variability among quantitative measurements provided by different commercial computerized tools should be considered.

## Figures and Tables

**Table 1:** Description of computed based tool functionalities.

Functionalities	Thoracic VCAR	Myriam	InferRead
Quantitative data automatically divided by lobes	no	no	yes
Quantitative data automatically divided by lung	yes	yes	yes
Total quantitative data	yes	yes	yes
Ability to segment manually	yes	no	no
Preliminary possibility of excluding automatically vascular structures	no	no	no
Preliminary possibility of excluding automatically airways	yes	yes	no
Possibility to create as many threshold windows as desired	yes	no	no
Possibility to modify the HU values in the threshold windows	yes	yes	no
Possibility to change the colors of the threshold windows	yes	yes	no
Possibility of 3D reconstruction	yes	yes	no
CE marking for lung study for COVID-19	no	yes	no
CE marking for lung study	yes	yes	no
Evaluation of emphysematous areas distinct from GGO, consolidation and residual parenchyma	yes	no	no
Possibility to evaluate GGO areas distinct from others	yes	yes	no
Possibility to evaluate consolidation areas distinct from others	yes	yes	no
Possibility to evaluate healthy parenchyma areas distinct from others	yes	yes	yes
Evaluation separately pleural effusion	no	no	no
Combined structured report	no	yes	no
Ability to export values to an unstructured report	yes	yes	yes
Automatic comparison of the previous exam with the current one in the follow-up	no	yes	yes

**Table 2:** The intraclass coefficient (ICC) among quantitative volumes obtained using different commercial computerized tools.

Variability	ICC	Lower Bound	Upper Bound
Total LHP (%)	0.17	0.05	0.31
Total GGO (%)	0.51	0.30	0.67
Total Consolidation (%)	0.20	0.04	0.37

Note. ICC = intraclass coefficient.

**Table 3:** Spearman correlation coefficient among quantitative volumes obtained using different tools.

		ThoracicVCAR Total LHP (%)	ThoracicVCAR Total GGO (%)	ThoracicVCAR Total Consolidation (%)	Myrian Total LHP (%)	Myrian Total GGO (%)	Myrian Total Consolidation (%)	Infervision Total LHP (%)	Infervision Total GGO (%)	Infervision Total Consolidation (%)
ThoracicVCAR Total LHP (%)	Spearman Correlation Coefficient	1.00	−0.964 **	−0.722 **	0.753 **	−0.677 **	−0.767 **	0.10	−0.499 **	−0.400 **
	*p*-value		0.00	0.00	0.00	0.00	0.00	0.39	0.00	0.00
ThoracicVCAR Total GGO (%)	Spearman Correlation Coefficient	−0.964 **	1.00	0.619 **	−0.780 **	0.741 **	0.748 **	−0.06	0.539 **	0.343 **
	*p*-value	0.00		0.00	0.00	0.00	0.00	0.60	0.00	0.00
ThoracicVCAR Total Consolidation (%)	Spearman Correlation Coefficient	−0.722 **	0.619 **	1.00	−0.536 **	0.557 **	0.559 **	−0.13	0.333 **	0.421 **
	*p*-value	0.00	0.00		0.00	0.00	0.00	0.26	0.00	0.00
Myrian Total LHP (%)	Spearman Correlation Coefficient	0.753 **	−0.780 **	−0.536 **	1.00	−0.935 **	−0.870 **	0.14	−0.570 **	−0.371 **
	*p*-value	0.00	0.00	0.00		0.00	0.00	0.20	0.00	0.00
Myrian Total GGO (%)	Spearman Correlation Coefficient	−0.677 **	0.741 **	0.557 **	−0.935 **	1.00	0.749 **	−0.10	0.568 **	0.314 **
	*p*-value	0.00	0.00	0.00	0.00		0.00	0.39	0.00	0.00
Myrian Total Consolidation (%)	Spearman Correlation Coefficient	−0.767 **	0.748 **	0.559 **	−0.870 **	0.749 **	1.00	−0.232 *	0.613 **	0.492 **
	*p*-value	0.00	0.00	0.00	0.00	0.00		0.04	0.00	0.00
Infervision Total LHP (%)	Spearman Correlation Coefficient	0.10	−0.06	−0.13	0.14	−0.10	−0.232 *	1.00	−0.462 **	−0.462 **
	*p*-value	0.39	0.60	0.26	0.20	0.39	0.04		0.00	0.00
Infervision Total GGO (%)	Spearman Correlation Coefficient	−0.499 **	0.539 **	0.333 **	−0.570 **	0.568 **	0.613 **	−0.462 **	1.00	0.601 **
	*p*-value	0.00	0.00	0.00	0.00	0.00	0.00	0.00		0.00
Infervision Total Consolidation (%)	Spearman Correlation Coefficient	−0.400 **	0.343 **	0.421 **	−0.371 **	0.314 **	0.492 **	−0.462 **	0.601 **	1.00
	*p*-value	0.00	0.00	0.00	0.00	0.00	0.00	0.00	0.00	

Note. LHP = lung healthy parenchyma, GGO = ground-glass opacity. ** The correlation is significant at the 0.01 level (two-tailed). * The correlation is significant at 0.05 level (two-tailed).

**Table 4:** ThoracicVCAR quantitative results compared with radiological findings.

		ThoracicVCAR LHP DX (%)	ThoracicVCAR LHP SX (%)	ThoracicVCAR Total LHP (%)	ThoracicVCAR GGO DX (%)	ThoracicVCAR GGO SN (%)	ThoracicVCAR Total GGO (%)	ThoracicVCAR Consolidation DX (%)	ThoracicVCAR Consolidation SX (%)	ThoracicVCAR Total Consolidation (%)
Overall radiological score ≤ 2	Median	86.60	84.50	85.80	10.90	12.00	10.30	0.70	0.70	0.70
	Minimum	59.70	65.40	67.90	2.10	3.10	2.90	0.10	0.20	0.20
	Maximum	96.70	95.30	95.60	30.70	28.20	25.90	3.50	3.20	2.30
Overall radiological score 3–4	Median	77.40	75.10	77.10	16.60	17.70	16.40	0.90	0.90	0.90
	Minimum	6.30	26.30	29.10	5.60	2.40	5.20	0.30	0.30	0.30
	Maximum	92.70	94.30	93.60	63.30	66.60	64.10	17.30	17.10	17.20
Overall radiological score 5–6	Median	67.90	64.80	66.00	26.00	27.90	26.80	1.40	1.30	1.30
	Minimum	35.50	42.30	42.60	6.20	5.90	6.00	0.30	0.20	0.30
	Maximum	90.60	90.90	90.80	40.50	46.30	40.50	16.40	5.80	10.70
Overall radiological score 7–8	Median	50.80	53.90	55.90	39.10	27.10	33.00	1.80	1.60	1.60
	Minimum	18.50	6.40	21.90	29.00	20.10	27.80	0.50	0.60	0.50
	Maximum	63.10	76.50	66.40	62.10	62.10	59.40	7.70	11.10	5.40
*p*-value at Kruskal–Wallis test		0.00	0.00	0.00	0.00	0.00	0.00	0.24	0.07	0.24
Spearman Correlation Coefficient		-0.76	-0.57	-0.74	0.68	0.58	0.65	0.40	0.38	0.39
*p*-value of Spearman Correlation		0.00	0.00	0.00	0.00	0.00	0.00	0.00	0.00	0.00

Note. LHP = lung healthy parenchyma; GGO = ground-glass opacity.

**Table 5:** Myrian quantitative results compared with radiological findings.

		Myrian LHP DX (%)	Myrian LHP SX (%)	Myrian Total LHP (%)	Myrian GGO DX (%)	Myrian GGO SN (%)	Myrian Total GGO (%)	Myrian Consolidation DX (%)	Myrian Consolidation SX (%)	Myrian Total Consolidation (%)
Overall radiological score ≤ 2	Median	69.10	66.80	67.00	26.20	27.30	26.70	4.00	4.70	4.70
	Minimum	22.70	23.90	25.80	9.10	9.50	9.30	2.10	2.10	2.20
	Maximum	88.30	88.10	88.20	61.90	71.30	62.00	14.80	15.60	13.70
Overall radiological score 3–4	Median	56.80	57.10	56.90	31.40	31.30	31.80	7.00	8.00	7.60
	Minimum	11.10	8.10	9.70	2.40	4.50	3.40	0.00	2.00	1.20
	Maximum	97.60	92.70	95.30	74.50	79.20	76.80	32.80	46.80	43.60
Overall radiological score 5–6	Median	38.60	40.30	41.00	44.00	44.25	43.05	13.10	12.70	13.95
	Minimum	13.70	13.90	16.90	18.50	19.40	18.90	2.60	2.50	2.60
	Maximum	78.50	77.80	78.20	69.10	68.60	68.90	29.60	32.90	31.10
Overall radiological score 7–8	Median	24.45	31.95	27.70	45.10	48.70	47.25	23.80	14.90	23.00
	Minimum	9.80	2.00	8.00	30.00	36.00	34.30	7.40	4.10	5.80
	Maximum	59.60	59.50	54.60	69.20	63.00	61.80	44.60	49.00	36.50
*p* value at Kruskal–Wallis test		0.00	0.00	0.00	0.00	0.00	0.00	0.00	0.00	0.00
Spearman Correlation Coefficient		−0.62	−0.55	−0.70	0.55	0.54	0.56	0.72	0.54	0.72
*p* value of Spearman Correlation		0.00	0.00	0.00	0.00	0.00	0.00	0.00	0.00	0.00

Note. LHP = lung healthy parenchyma, GGO = ground glass opacity.

**Table 6:** InferRead quantitative results compared with radiological findings.

		InferRead Total LHP (%)	InferRead Total GGO (%)	InferRead Total Consolidation (%)	−570/−470 (%)	−470/−370 (%)	−370/−270 (%)	−270/−170 (%)	−170/−70 (%)	−70/30 (%)	30/60 (%)	OTHER (%)
Overall radiological score ≤ 2	Median	66.63	26.19	4.80	11.20	8.40	5.72	3.86	2.66	1.40	0.23	61.89
	Minimum	0.00	0.00	0.00	0.00	0.00	0.00	0.00	0.00	0.00	0.00	0.00
	Maximum	96.08	49.88	33.74	19.82	17.69	14.88	13.78	12.54	19.27	5.68	95.90
Overall radiological score 3–4	Median	60.39	28.62	5.83	13.55	9.07	6.93	4.96	3.23	2.30	0.39	53.94
	Minimum	0.00	0.00	0.00	0.00	0.00	0.00	0.00	0.00	0.00	0.00	0.00
	Maximum	87.41	47.36	40.72	20.60	17.48	15.89	11.53	9.93	21.63	11.50	86.27
Overall radiological score 5–6	Median	57.44	32.39	8.09	12.90	9.99	8.07	7.02	4.78	3.56	0.48	50.08
	Minimum	0.00	0.00	0.00	0.00	0.00	0.00	0.00	0.00	0.00	0.00	0.00
	Maximum	80.98	47.40	23.74	18.63	16.93	14.63	11.88	9.31	12.38	2.05	78.96
Overall radiological score 7–8	Median	39.95	39.80	10.16	12.01	13.23	12.20	10.59	7.34	3.77	0.56	28.76
	Minimum	32.87	10.86	1.43	6.18	2.92	1.76	0.93	0.64	0.68	0.11	11.27
	Maximum	86.74	58.12	27.35	19.24	21.38	26.91	21.60	12.34	13.34	1.71	85.47
*p*-value at Kruskal–Wallis test		0.00	0.00	0.03	0.00	0.00	0.00	0.00	0.00	0.13	0.75	0.00
Spearman Correlation Coefficient		−0.08	0.38	0.37	0.26	0.38	0.40	0.41	0.38	0.36	0.32	−0.08
*p*-value of Spearman Correlation		0.41	0.00	0.00	0.01	0.00	0.00	0.00	0.00	0.00	0.00	0.39

Note. LHP = lung healthy parenchyma; GGO = ground-glass opacity.
